# Augmented Dried versus Cryopreserved Amniotic Membrane as an Ocular Surface Dressing

**DOI:** 10.1371/journal.pone.0078441

**Published:** 2013-10-30

**Authors:** Claire L. Allen, Gerry Clare, Elizabeth A. Stewart, Matthew J. Branch, Owen D. McIntosh, Megha Dadhwal, Harminder S. Dua, Andrew Hopkinson

**Affiliations:** Ophthalmology, Division of Clinical Neuroscience, University of Nottingham, Nottingham, United Kingdom; University of Missouri-Kansas City, United States of America

## Abstract

**Purpose:**

Dried amniotic membrane (AM) can be a useful therapeutic adjunct in ophthalmic surgery and possesses logistical advantages over cryopreserved AM. Differences in preservation techniques can significantly influence the biochemical composition and physical properties of AM, potentially affecting clinical efficacy. This study was established to investigate the biochemical and structural effects of drying AM in the absence and presence of saccharide lyoprotectants and its biocompatibility compared to cryopreserved material.

**Methods:**

AM was cryopreserved or dried with and without pre-treatment with trehalose or raffinose and the antioxidant epigallocatechin (EGCG). Structural and visual comparisons were assessed using electron microscopy. Localisation, expression and release of AM biological factors were determined using immunoassays and immunofluorescence. The biocompatibility of the AM preparations co-cultured with corneal epithelial cell (CEC) or keratocyte monolayers were assessed using cell proliferation, cytotoxicity, apoptosis and migration assays.

**Results:**

Drying devitalised AM epithelium, but less than cryopreservation and cellular damage was reduced in dried AM pre-treated with trehalose or raffinose. Dried AM alone, and with trehalose or raffinose showed greater factor retention efficiencies and bioavailability compared to cryopreserved AM and demonstrated a more sustained biochemical factor time release *in vitro*. Cellular health assays showed that dried AM with trehalose or raffinose are compatible and superior substrates compared to cryopreserved AM for primary CEC expansion, with increased proliferation and reduced LDH and caspase-3 levels. This concept was supported by improved wound healing in an immortalised human CEC line (hiCEC) co-cultured with dried and trehalose or raffinose membranes, compared to cryopreserved and fresh AM.

**Conclusions:**

Our modified preservation process and our resultant optimised dried AM has enhanced structural properties and biochemical stability and is a superior substrate to conventional cryopreserved AM. In addition this product is stable and easily transportable allowing it to be globally wide reaching for use in clinical and military sectors.

## Introduction

Amniotic membrane (AM) is the inner most extraembryonic membrane that surrounds the foetus in a sac of amniotic fluid, functioning as a protective barrier to ascending infection and trauma during pregnancy[1], [2]. This is separated from a single layer of cuboidal epithelium by a basement membrane (BM). The epithelium is metabolically active, maintaining amniotic fluid homeostasis and secreting embryonic stem cell factors[3].

AM has proven to be a highly versatile surgical adjunct. The clinical use of AM was first reported in the treatment of skin wounds, in 1910[4], and over the last century its application for a variety of conditions has become widespread[5]–[10].

Therapeutic AM is extensively used in ophthalmic surgery and was first applied with chorion, as a replacement for scarred conjunctival tissue[11]. AM is now commonly used as a permanent graft or a temporary patch in a plethora of conjunctival and corneal procedures[12]–[16].

AM has been shown to act as a scaffold for cell growth[17], promote epithelial wound healing[18] and to exert anti-inflammatory[19], anti-angiogenic[20], anti-fibrotic[21] and anti-microbial effects[22]. These mechanisms are, in part, attributed to a wide range of biological factors present in AM, for example epidermal growth factor (EGF) and transforming growth factor (TGF)-β1[23].

In many countries, AM is obtained from elective caesarean section deliveries and typically frozen in medium containing glycerol or dimethylsulphoxide (DMSO) while the donor is screened for a spectrum of infectious diseases. The effects of DMSO and glycerol preservatives on the structural and biochemical integrity of cryopreserved AM are unclear[24]. Following freezing AM is considered non-viable and following thawing soluble factors presumed to be beneficial are extensively depleted from the tissue, potentially reducing its efficacy. Wolbank *et al* [25] has additionally shown that there are significantly lower levels of angiogenic factors in cryopreserved AM compared to fresh but they did not assess lyophilised AM. A number of studies have reported extensive depletion of soluble factors, presumed to be beneficial, from cryopreserved AM[26]–[28]. While frozen preparations of AM account for the majority of procedures, dried preparations have gained popularity as substrates for epithelial growth during ocular surface reconstruction[29], [30], to treat corneal perforations and leaks[31] and pterygium[32]. Moreover, as dried preparations can be kept at room temperature, they eliminate the need for a cold chain and are therefore suitable for use in developing countries and in military environments.

Conventional freeze-drying requires the tissue to be frozen prior to drying. In AM, this appears to result in structural freeze damage and subsequent factor loss as compared with conventional cryopreservation techniques[32]. Since AM is typically less than 100 microns thick, it is possible to dry it in a freeze-dryer vacuum without the pre-freeze step, and this is herein referred to as dried AM.

Sterile, dried AM can be prepared in the presence of complex saccharide lyoprotectants such as trehalose[33], a non-reducing disaccharide and a major energy source for some organisms during anhydrobiosis[34]. Trehalose replaces intracellular water during dehydration or freezing to form a glassy matrix, thus preventing disruption of internal cell organelles[35]. Trehalose is regarded as an exceptional lyoprotectant due to its high thermostability, a wide pH-stability range, high water retention capabilities, and its non-toxicity[36] and has been used to protect a plethora of cell types during freeze-drying[37]–[40]. An alternative lyoprotectant, the trisaccharide raffinose accumulates in organelles during extreme exposure[41] and acts as a free radical scavenger at high temperatures[42]. A combination of raffinose and glutamine has shown to be effective in preserving sperm acrosomes, facilitating freezability[43]. The potent antioxidant epigallocatechin gallate (EGCG) in combination with trehalose has been shown to promote the viability of mononuclear cells and maintain intact plasma membranes following freeze-thawing and freeze-drying[44].

In this study, we investigated the structural and biological properties of dried AM, and its biocompatibility as an ocular surface dressing, using cryopreserved AM as a comparator. In addition we pre-treated AM with trehalose or raffinose and EGCG to further augment the tissue quality.

## Materials and Methods

### Tissue procurement and preparation

The following research was carried out with the approval of the Nottingham Research Ethics Committee and the study (OY110101) complied with the tenets of the Declaration of Helsinki. Written informed consent from the donor (participant age range 25–37) or the next of kin was obtained for sample use in this research project and prepared according to previously published methodology[45]. Patients with a history of antenatal problems e.g. gestational diabetes or placenta praevia were excluded from the study. In brief, excess blood was washed away with a balanced salt solution and the amnion was separated from the placenta and the chorion. The amnion was further washed for 3×15 minutes to allow the spongy layer to expand for easy removal. Mid-region sections were preserved and then circular sections were prepared using a 5 cm Ø trephine. Sections were used immediately or placed in vacuum pouches prior to drying and vacuum sealing.

### Tissue preservation optimisation

To optimise the preservation process, prior to drying, AM segments were uniformly spread out in Petri dishes and bathed in a series of solutions containing glycerol/PBS (ratios of 1∶4 and 1∶2), DMSO (Sigma-Aldrich)/phosphate buffered saline (PBS, 5 and 10% v/v), and tertiary butyl alcohol (TBA, Sigma-Aldrich)/PBS (10 and 40% v/v) for 10 minutes each. Alternatively segments were incubated with 2, 10, and 25% w/v D- (+)-Trehalose dihydrate (Acros Organics, Belgium) in PBS or 25, 100 and 200 mM w/v D-(+)-Raffinose pentahydrate (Acros Organics), and EGCG (1 mg/mL, Sigma-Aldrich) for 2 hours at 37°C.

### Optimised preservation parameters

Processed AM segments from 12 donors were either used fresh or prepared and preserved using one of five defined methodologies.

#### Cryopreserved

AM segments were preserved in sterile Dulbecco's PBS (Sigma-Aldrich) in 20 mL sterile tubes, at −80°C, using established methodologies within our department[45].

#### Dried

Fresh segments were uniformly spread out, epithelial side up, in vacuum pouches. This was then double heat sealed on three sides prior to drying. Drying was performed using an Alpha 1–4 LSC freeze-dryer (Christ, Germany). Prior to sample drying the ice condenser was equilibrated to −45°C. This allows any vapour present in the drying chamber to be removed during the drying procedure, by freezing to the condenser itself. The drying cycle comprised of a main dry phase for 1 hour (shelf temperature 15°C, vacuum pressure 1.030 mbar, safety pressure 1.650 mbar) followed by a final drying phase for 30 minutes (shelf temperature 20°C, vacuum pressure 0.0010 mbar, safety pressure 1.650 mbar). Following completion of the drying cycle the pouch was heat sealed under vacuum using a Multiple 315 vacuum packaging chamber, (Orved, Italy) and stored at room temperature, and away from direct light, until further analysis.

#### Denuded

AM segments were denuded of epithelium with thermolysin (Sigma-Aldrich, 125 µg/mL in PBS) according to our published methodologies[45] and dried as previously described.

#### Trehalose

AM was pre-treated with 10% w/v D- (+)-Trehalose dihydrate and EGCG (1 mg/mL for 2 hours at 37°C. Prior to drying membrane sections were washed briefly in a 1∶10 dilution of the original trehalose solution to remove excess residue from the surface.

#### Raffinose

AM was pre-treated with 100 mM D-(+)-Raffinose pentahydrate and EGCG (1 mg/mL) for 2 hours at 37°C. Prior to drying membrane sections were washed briefly in a 1∶10 dilution of the original raffinose solution to remove excess residue.

### Tissue transparency

Transparency of the pre-treated and dried membranes were assessed by taking images against printed material and a dark granulated background and compared to non-treated dried only control material. Membranes were graded according to a 3 point scale with control material being assigned the maximum of 3 for optimal transparency; this was carried out by two separate investigators.

### Electron microscopy (EM)

Scanning EM (SEM) and transmission EM (TEM) studies were performed on membranes from 3 separate donors, prepared in duplicate following different preservation techniques. For each sample, 1 cm diameter discs of preserved AM samples were overlaid on corresponding discs of polyvinylidene fluoride (PVDF) membrane (epithelial side up) and processed for SEM and TEM according to previously published methodologies[45]. SEM samples were observed in a JEOL 840 microscope (JEOL, UK) and appropriate digital images were recorded using an integrated Iscan digital imaging system. A JEOL 1010 microscope was used to observe the TEM sections at 100 kV, and recorded using an SIS integrated digital camera system.

### Biochemical analysis

Soluble proteins were extracted from segments of fresh, cryopreserved, denuded, dried, trehalose or raffinose treated membranes from 3 separate donors. Samples were ground under liquid nitrogen and reconstituted in 1× Tris buffered saline +0.05% v/v Triton-X (Sigma-Aldrich) (TBS_TX_) buffer for 20 minutes at room temperature. Insoluble material was removed by centrifuging at 20,000*×g*, for 15 minutes, at 4°C and the protein concentration of each supernatant was determined using a 2D Quant kit (GE Healthcare, UK). Protein arrays were carried out in duplicate using SearchLight immunoassay technology (Aushon Biosystems, USA) for a profile of 48 protein analytes. Analytes failing to provide a data value were omitted from the study and remaining data normalised according to ng/mg of protein extract.

### ELISA

Differently preserved AM sections (5 cm Ø) from 3 separate donors were washed in 5 mL saline containing protease inhibitors (complete protease inhibitor tablets; Roche, UK) for 3×10 minutes. Cryopreserved AM storage medium and washes were concentrated and retained for analysis. Samples were concentrated using Amicon® centrifugal filter devices (MWCO 10 kDa, Millipore, UK) and protein concentrations determined as previously described. EGF and TGF-β1 concentrations were determined using ELISA duo kits (R & D Systems, UK) with microplates pre-coated with monoclonal antibodies specific for the human markers in question, as per manufacturer's instructions. Sample absorbances were read at 450 nm and concentrations were then calculated from a standard curve of known values, and subsequently normalised against protein concentration.

### Biochemical stability

Triplicate AM sections from 3 donor samples were preserved as previously described and stored in sterile vacuum pouches at ambient temperature and away from direct light, for a period of 4, 8, 12, 24, 48 and 60 weeks. Following storage, soluble proteins were extracted from the tissue sections and protein concentrations determined using a 2D Quant kit and EGF and TGF-β1 concentrations assessed using ELISA kits previously described.

### Immunofluorescence

AM sections from 3 donors for each sample preservation type were prepared and immunostained according to published methodologies[45]. Briefly, AM sections were out-spread, epithelial side up on a flat surface, a layer of optimal cutting temperature (OCT) freezing compound (Leica, Germany) applied to the surface and covered with a piece of microscope tissue. Using the microscope tissue, the amnion was repeatedly folded (5–7 mm per fold) ensuring any air bubbles between folds were expelled and 1 cm sections of the folded tissue section were carefully placed vertically into pre-moulded aluminium foil cups (1.5 cm in height) containing pre-chilled OCT compound and immediately frozen using liquid nitrogen. Once frozen the samples were either stored at −80°C or 6 µm sections were prepared using a cryostat (Leica), blocked and directly stained with primary antibodies, overnight, at 4°C (Table 1). Primary antibodies were detected using secondary anti-mouse (A11029) or anti-rabbit (A21430), both from Invitrogen, Paisley, UK) fluorophore conjugates applied at 1∶400 and incubated for 1 hour at room temperature. Slides were counterstained with 4',6-Diamidino-2-phenylindole (DAPI; 1.25 µg/mL; Santa Cruz, Germany). For each staining run and each antibody, appropriate positive controls (corneal tissue sections) and negative controls (in which non-immune immunoglobulin was substituted for the primary antibody) were performed to ensure quality control. Slides were examined on a fluorescence microscope (Olympus BX51) and imaged using CellˆF software (Olympus, UK). Each experiment was performed in triplicate.

**Table 1:** A summary of primary antibodies used for immunofluorescent analysis.

Target	Source	Clone	Species Raised	Dilution Factor
BDNF	abcam^a^	ab72439	rabbit	1∶20
EGF	R & D^b^	MAB236	mouse	1∶20
E-Selectin	abcam	ab6630	mouse	1∶100
HGF	R & D	AF-294-NA	goat	1∶7
ICAM-1	abcam	ab20	mouse	1∶20
IL-8	abcam	ab89336	mouse	1∶20
KGF	abcam	ab9598	rabbit	1∶1000
MMP2	abcam	ab7032	mouse	1∶500
MMP3	abcam	ab18898	mouse	1∶33
MMP9	abcam	ab51203	mouse	1∶250
β-NGF	abcam	ab6199	rabbit	1∶100
PEDF	abcam	ab14993	mouse	1∶20
TGF-β1	R & D	MAB240	mouse	1∶25
TSP-1	abcam	ab1823	mouse	1∶100

Abbreviations: BDNF, brain derived neurotrophic factor; HGF, hepatocyte growth factor; ICAM-1, intercellular adhesion molecule-1; IL-8, interleukin-8; KGF, keratinocyte growth factor; MMP, matrix metalloprotease; NGF, nerve growth factor; PEDF, pigment epithelium derived factor; TGF, transforming growth factor; TSP, thrombospondin.

a abcam, UK.

b R & D Systems, Oxfordshire, UK.

### Primary corneal epithelial cell (pCEC) isolation and culture

Cells were isolated according to a previously published methodology[46]. In brief corneoscleral rims remaining after penetrating keratoplasty from consented donors were processed for culture within 5 days. Excess sclera was removed and posterior stroma and endothelium stripped away. The rims were divided into two approximately eight sections and placed epithelial side up, on Nunclon 35×10 mm culture plates (VWR international Ltd, UK). pCEC were cultured in CnT-BM.1 basal culture medium containing CnT-20.A, B and C supplements (CellnTec, Switzerland), 2.5 µg/mL Plasmocin™ (Autogen Bioclear, UK), and 0.02 µg/mL gentamicin, 0.5 ng/mL amphotericin B (combination, Gibco, Invitrogen, UK). Explants were removed and placed into new culture plates once a confluent epithelial sheet was evident.

### Primary keratocyte (pKer) isolation and culture

Keratocytes were isolated according to a previously published methodology[47]. In brief, the epithelial and endothelial layers were removed by mechanical scraping, and the remaining limbal tissue was divided into small pieces and digested in 0.1 mg/ml collagenase type IA (Sigma Aldrich, UK). The tissue was incubated for approximately 18 hours at 37°C, 5% v/v CO_2_ and filtered with a 41-µm nylon filter (Fisher Scientific, UK), to remove non cellular debris. Culture medium was added to the collagenase filtrate solution prior to centrifugation at 450*×g* for 6 minutes. The cell pellet was resuspended in M199 basal culture medium (Sigma-Aldrich) supplemented with 20% v/v heat-inactivated FBS (Fisher Scientific), 2.5 µg/ml Plasmocin™, 0.02 µg/ml gentamicin, 0.5 ng/ml amphotericin B, and 1.59 mM L-glutamine (Sigma-Aldrich).

### Corneal epithelial cell line culture

hiCEC (immortalised human corneal epithelial cells, passages 19–26; a kind donation from Araki-Sasaki, Japan[48]) were expanded in EpiLife basal culture medium (Invitrogen, UK) supplemented with 20% v/v FBS, 2.5 µg/ml Plasmocin™, 0.02 µg/ml gentamicin and 0.5 ng/ml amphotericin B.

Cell cultures were maintained at 37°C under 5% v/v CO_2_, replacing culture medium every 2–3 days until confluent. Cells were passaged at 80% confluence at a 1∶3 ratio.

### *In vitro* cell culture model

Differently preserved AM sections were cultured directly or indirectly with hiCEC, pCEC and keratocytes. Indirect cultures were constructed using 24-well CellCrown™ inserts (Scaffdex, Finland). 15 mm membrane discs were laid over the support and held in place with an outer ring. The support was then inverted in a 24-well plate so it was immersed in the media but not in direct contact with the cells. Direct cultures were assembled by seeding the cells directly on top of the AM/CellCrown™ supports. Cells were seeded at 0.05×10^6^ and maintained at 37°C under 5% v/v CO_2_ for 3 days and then used in the following assays:

#### Biochemical release

Factor time release studies were carried out using the above system apart from the membranes were submersed in sterile PBS. Samples of PBS (120 µl) were taken following 1, 2, 4 and 10 days in culture. Samples were stored at −80°C prior to SearchLight protein array analysis and EGF and TGF-β1 ELISA experiments.

#### Biocompatibility assays

Cell proliferation was assessed using the *in vivo* Cell-8 assay (Sigma-Aldrich), cytoxicity was measured using a lactate dehydrogenase (LDH) enzyme based assay (Roche Diagnostics Ltd, UK) and apoptosis was determined using a caspase-3 colorimetric assay (R & D Systems, UK). All assays were performed according to manufacturer's protocols. Levels were calculated by subtracting cell only control values for each day and results are expressed as a percentage increase or decrease relative to the previous day.

#### Cell migration assay

A scratch wound closure assay was performed five days post seeding, on confluent cultures starved of serum and EGF for 24 hours. A standard single linear scratch with a defined length of 1.6 cm was created in the cell monolayers across each well using a 10 µl pipette tip, giving a 300 µm wound width. Unattached cells were washed away and medium was replaced with media containing EGF and FBS. Wounds were photographed immediately (day 0) and then 2, 4, 6 and 10 days, at four pre-determined positions by phase-contrast imaging at 100× magnification. Wound healing for each culture was reported as the average linear speed of the wound edge closure over a 10 day period, using ImageJ software (Wayne Rasband, National Institute of Health).

### Statistical Analysis

Results are presented as mean ± SEM. Statistical analyses were performed using the nonparametric Mann-Whitney *U* test and p<0.05 was considered significant.

## Results

### Preservation of amnion

Visual assessment of the differently preserved AM substrates, summarised in Table 2, revealed dried AM in the absence of a lyoprotectant produced a thin, furrowed and papery biomaterial. Pre-treatment with the saccharide lyoprotectants produced a denser membrane with powdery areas of residual sugar post drying. However incorporating a 1∶10 dilution wash of the original lyoprotectant removed residual sugar deposits present and increased transparency to a level observed with dried only. Pre-treatment with glycerol or TBA produced membranes with increased brittleness and greasiness and reduced transparency compared to no pre-treatment. Similar results were observed with DMSO.

**Table 2:** A summary of preservation optimisation strategies employed and their impact on membrane transparency.

Lyoprotectant/Conditions	General Membrane Observations	Transparency
Control (VDAM Only)	thin/furrowed/papery	+++
Glycerol 25% v/v (4°C)	thin/furrowed/papery/sticky	+
Glycerol 50% v/v (4°C)	thin/furrowed/papery/sticky	+
DMSO 5% v/v (−80°C)	thin/furrowed/papery/fragile	++
DMSO 10% v/v (−80°C)	thin/furrowed/papery/fragile	++
TBA 10% v/v RT	thinner/more uniform/fragile	+
TBA 40% v/v RT	thinner/dehydrated/less uniform/fragile	+
Trehalose 10% w/v (2 hr/37°C)	thicker/more uniform/trehalose residue	++
Trehalose 10% w/v+TBA 10% v/v (2 hr/37°C)	thicker/more uniform/trehalose residue	++
Raffinose 100 mM w/v (2 hr/37°C)	thicker/more uniform/raffinose residue	++
As above +1∶10 raffinose wash (2 hr/37°C)	Thicker/more uniform/reduced residue	+++

Membranes were incubated in the following solutions for 10 minutes prior to drying and tissue transparencies were graded in comparison to a non-treated, dried only control.

Abbreviations: DMSO, dimethyl sulphoxide; TBA, tertiary butyl alcohol; RT, room temperature. All concentrations are represented as v/v except raffinose w/v.

### Effects of preservation techniques on AM structure

**Figure 1:**
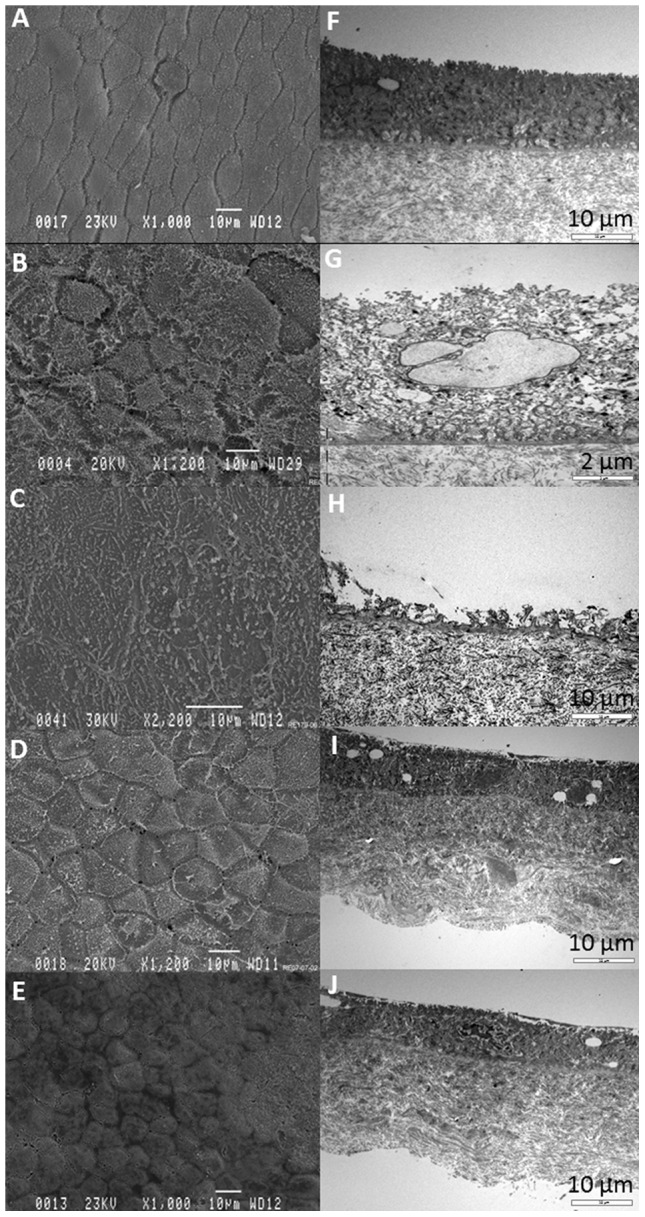
SEM and corresponding TEM micrographs of the epithelial and stromal layers in preserved AM substrates. Fresh, (**A**, **F**); cryopreserved, (**B**, **G**); denuded (**C**, **H**); dried (**D**, **I**) and post treatment with trehalose (**E**, **J**). Micrographs depict extensive damage to the AEC layer and microvilli post cryopreservation compared to fresh, dried and trehalose treated substrates. Images shown are representative of triplicate experiments carried out on three donor membranes.

Following cryopreservation the typical amniotic epithelial cell (AEC) polygonal shape and cell patterns were lost. AEC appeared mainly intact but heavily damaged with clear fissures present between the cells with the damage and absence of surface microvilli (Figure 1B). AEC had been clearly removed from the denuded sample, exposing a uniform and consistent BM surface (Figure 1C). The appearance of AM following pre-treatment with trehalose (Figure 1E) and drying was more akin to fresh AM, with a more polygonal AEC structure and with limited surface and microvillus damage compared to drying alone (Figure 1D). Representative TEM images show a disorganised and vacuous epithelial cell layer in cryopreserved material (Figure 1G) compared to fresh AM (Figure 1F). Following drying the epithelial and stromal layers appeared much more compact with dense collagen networks (Figures 1I & J) but with little evidence of damage compared to cryopreserved AM.

### The effects of optimised preservation on biochemical bioavailability

To characterise the potential susceptibility of each factor to different preservation conditions, data from the SearchLight protein arrays (Table 3) was interpreted according to potential *in situ* localisation and solubility state. This generated 5 specific sub-divisions of cohorts; (i) epithelial factors that were predominantly soluble, reduced with epithelial damage and therefore susceptible to preservation related stripping and removed with denuding; (ii) epithelial, predominantly insoluble factors not reduced by preservation but removed with denuding; (iii) stromal factors that were partially soluble, reduced by preservation but not by denuding; (iv) stromal and predominantly insoluble factors not reduced by preservation and proportionately increased following denuding and (v) factors that were undefined and considered to be both epithelial and stromal. Of the 45 protein factors analysed (Table 3) there was an even divide between epithelial 47% (21/45) and stromal 44% (20/45)-related factors, with 9% (4/45) of factors undefined. Analysis of the epithelial cohort showed that 43% (9/21) of factors were soluble and 57% (12/21) were bound. Within the stromal cohort 35% (7/20) of factors were predominantly soluble and 65% (13/20) were bound and in-soluble.

**Table 3:** SearchLight protein array expression profiles of preserved AM substrates.

Factor	Fresh (ng/mg TP)	Denuded (ng/mg TP)	Cryopreserved (ng/mg TP)	Dried (ng/mg TP)	Raffinose (ng/mg TP)
**Epithelial/Soluble**					
**hAng2**	*0.005*±*0.005* †	**0.000**±**0.000** *	**0.000**±**0.0** *	*0.017*±*0.018* †	**0.000**±**0.000** *
**hFibrinogen**	*262.972*±*147.568* † #	**24.365**±**13.547** *	**112.80**±**48.600** *	***185.786***±***85.615*** * †	***224.246***±***99.004*** † #
**hMMP3**	*0.194*±*0.080* †	**0.000**±**0.000** *	**0.085**±**0.049** *	*0.308*±*0.178* †	*0.383*±*0.187* †
**hBNGF**	*0.003*±*0.001*	***0.002***±***0.001***	**0.001**±**0.000**	***0.002***±***0.001***	*0.003*±*0.001*
**hPEDF**	*85.643*±*55.701* † #	**0.000**±**0.000** *	**21.894**±**13.005** *	***28.400***±***25.919*** * †	*168.303*±*131.407* † #
**hSCF**	0.173±0.069#	**0.000**±**0.000** *	1.004±0.394#	**0.138**±**0.068**	*2.216*±*0.885* † #
**hTNFα**	*0.006*±*0.002* †	**0.000**±**0.000** *	**0.003**±**0.002**	***0.004***±***0.002***	**0.002**±**0.001**
**hTRAIL**	*0.104*±*0.044* † #	**0.009**±**0.004** *	**0.065**±**0.038** *	***0.090***±***0.035*** †	***0.098***±***0.044***
**hVCAM1**	*18.405*±*9.258* #	**0.000**±**0.000** *	**13.150**±**5.076** #	**9.709**±**5.781** *	*23.206*±*11.844* † ^#^
**Epithelial/Insoluble**					
**hCNTF**	0.034±0.013	**0.000**±**0.000** *	0.700±0.486#	0.040±0.020	0.269±0.104#
**hFASL**	*0.003*±*0.000* †	**0.000**±**0.000** *	**0.000**±**0.000** *	*0.003*±*0.002* †	**0.000**±**0.000** *
**hIFNγ**	*0.001*±*0.000*	**0.000**±**0.000**	**0.000**±**0.0**	*0.001*±*0.001*	***0.000***±***0.000***
**hIL1β**	0.001±0.000	**0.000**±**0.000**	0.002±0.001	0.001±0.000	**0.000**±**0.000**
**hIL1rα**	229.968±100.232	**75.830**±**34.252** *	266.792±117.48	*301.567*±*139.481* †	***212.478***±***85.682***
**hMIF**	1762.011±713.587	**22.375**±**16.784** *	4383.564±1816.53#	2792.842± 1201.670	3961.489±1588.013#
**hMIP1α**	*0.078*±*0.032* †	**0.000**±**0.000** *	**0.038**±**0.017** *	*0.097*±*0.038* †	***0.077***±***0.036*** †
**hMIP1β**	0.023±0.011	**0.002**±**0.001** *	0.059±0.038	0.040±0.021	*0.063*±*0.026*
**hMMP8**	0.245±0.117	0.262±0.181	0.727±0.290#	0.580±0.346	0.460±0.219
**hNT3**	0.000±0.000	0.000±0.000	0.007±0.0040#	0.001±0.000	0.011±0.005† #
**hTGFα**	0.036±0.024	**0.006**±**0.003** *	0.049±0.025	*0.056*±*0.047*	0.044±0.021
**hTSP1**	*1440.505*±*659.289*	**0.000**±**0.000** *	**1241.654**±**565.711** *	***1325.606***±***762.415*** †	***1270.854***±***503.243*** *
**hE-Selectin**	*0.415*±*0.160* †	*0.312*±*0.145* †	**0.176**±**0.071** * ^♦^	*0.353*±*0.148* †	***0.283***±***0.110*** * †
**Stromal/Soluble**					
**hGDNF**	*0.016*±*0.019* † # ♦	*0.024*±*0.028* † #	**0.000**±**0.0** *	**0.000**±**0.000** *	**0.000**±**0.000** *
**hIL8**	*0.116*±*0.058* †	*0.144*±*0.081* †	**0.062**±**0.036** * ♦	***0.114***±***0.047*** †	*0.146*±*0.079* †
**hKGF**	*0.106*±*0.060* †	***0.095***±***0.082*** †	**0.049**±**0.022** * ♦	*0.106*±*0.045* †	*0.179*±*0.110* † #
**hMMP7**	0.248±0.103	**0.201**±**0.088**	0.390±0.250	0.280±0.126	*0.624*±*0.313* † #
**hTGFβ1**	*2.753*±*1.436* † #	***2.013***±***0.817*** †	**1.389**±**0.554** * ♦	***1.714***±***0.686*** * †	*6.760*±*4.865* †
**hTGFβ2**	0.469±0.212♦	*0.808*±*0.344*	0.505±0.249	*0.657*±*0.429* †	*0.706*±*0.370*
**Stromal/Insoluble**					
**hEGF**	*0.946*±*0.429* †	**0.584**±**0.401** *	**0.762**±**0.432** *	*1.123*±*0.560* †	*0.964*±*0.396* †
**hFGFbasic**	0.609±0.363# ♦	*2.35*±*1.559* † #	0.619±0.520# ♦	**0.172**±**0.068** * ♦	*1.467*±*0.891* † # ♦
**hHGF**	*34.004*±*14.493* † ♦	*84.912*±*35.142* † #	**5.313**±**2.051** * ♦	*62.169*±*24.638* † ♦	***6.087***±***2.366*** * ♦
**hHGH**	*0.811*±*0.444* † ♦	*1.306*±*1.181* †	**0.098**±**0.039** * ♦	*1.274*±*0.754* †	***0.183***±***0.095*** * † ^♦^
**hICAM1**	*8.830*±*3.573* ♦	*23.038*±*10.213* † #	**7.118**±**3.611** ♦	*11.770*±*5.444* † ♦	*13.357*±*6.568* † ♦
**hICAM3**	*2.149*±*0.911* †	*4.032*±*1.628* †	**0.679**±**0.364** *	*3.911*±*1.842* †	***0.759***±***0.293*** * ♦
**hIL1α**	0.047±0.021	*0.135*±*0.078* † #	0.073±0.028♦	0.066±0.030♦	***0.037***±***0.015***
**hMMP1**	0.285±0.201♦	*10.972*±*6.470* † #	0.361±0.242♦	*0.508*±*0.249* † ♦	*0.574*±*0.331* † ♦
**hMMP2**	7.705±3.035♦	*23.204*±*10.413* † #	8.080±3.364♦	**6.613**±**3.821** ♦	*13.905*±*5.639* † # ♦
**hMMP9**	1.308±0.523♦	*2.007*±*0.843* †	1.327±0.653♦	*1.749*±*0.709* †	**1.194**±**0.511** ♦
**MMP10**	0.820±0.326♦	*2.032*±*0.966* † #	1.000±0.407♦	0.986±0.472♦	*1.216*±*0.518* † ^#^ ^♦^
**hTIMP2**	*29.566*±*13.229* ♦	*44.787*±*19.990* † #	**20.719**±**9.060** ♦	*32.132*±*15.424* † ♦	*29.761*±*12.655* † ♦
**hVEGF**	*0.006*±*0.003* †	*0.007*±*0.004* †	**0.003**±**0.003**	***0.004***±***0.002***	*0.006*±*0.003* †
**hBDNF**	*1.493*±*0.587* † #	***0.372***±***0.195*** * †	**0.230**±**0.166** * ♦	***1.174***±***0.459*** †	**1.280**±**0.547** †
**hIL6**	0.018±0.008	**0.008**±**0.004** *	0.041±0.017#	0.028±0.012	0.038±0.003
**hRANTES**	0.066±0.031	*0.079*±*0.040*	0.066±0.037	*0.164*±*0.068* †	*0.121*±*0.060* †
**hTIMP1**	*97.548*±*47.431* † #	**14.037**±**7.062** *	**69.815**±**27.325** * #	**59.057**±**22.863** *	*120.546*±*60.531* † #

Soluble proteins were extracted from differently preserved AM samples in triplicate and protein arrays were carried out in duplicate using SearchLight immunoassay technology (Aushon Biosystems, USA).

Each data value represents the average value of duplicate analysis on 3 separate biological donors. Abbreviations: TP, Total Protein; Ang2, Angiotensin 2; FGF, fibroblast growth factor; TIMP, tissue inhibitor of metalloproteases; VEGF, vascular endothelial growth factor; FASL, Fas ligand; TRAIL, tumour necrosis factor-related apoptosis-inducing ligand; VCAM, vascular cellular adhesion molecule; RANTES, regulated upon activation, normal T-cell expressed and secreted; MIP, macrophage inflammatory protein; MIF, macrophage migration inhibitory factor; IFN, interferon; TNF, tumour necrosis factor; HBEGF, heparin binding EGF-like growth factor; HGH, human growth hormone; SCF, stem cell factor; TGF, transforming growth factor; NGF, nerve growth factor; CNTF, ciliary neurotrophic factor; GDNF, glial cell-derived neurotrophic factor; NT3, neurotrophin 3.

*significant decrease compared to fresh AM;

† significant increase compared to cryopreserved AM;

# significant increase compared to dried AM;

♦ significant decrease compared to denuded AM; bold type denotes a decrease in level compared to the level in fresh AM and italicised type denotes an increase in level compared to the level in cryopreserved AM.

To validate the SearchLight data, and to explore in more detail factor retention efficiencies, ELISA and *in situ* localisation experiments were performed for EGF and TGF-β1. There was a significant reduction in both EGF and TGF-β1 factor retention in cryopreserved AM compared to fresh, dried, trehalose and raffinose treated tissue. This pattern was most evident with the epithelial factor EGF, with cryopreserved AM retaining only 13% of total EGF overall and leaching 57% in the primary wash alone. Drying alone and in the presence of trehalose or raffinose and the antioxidant EGCG retained EGF at efficiencies of 61, 88 and 83% respectively (Table 4). The lyoprotectants provided greater factor retention and efficiencies similar to fresh AM. This was also observed in corresponding TGF-β1 factor retention profiles with cryopreserved AM retaining 71% compared to fresh (94%), dried (86%), trehalose (93%) and raffinose treated AM (92%) (Table 5).

**Table 4:** The effects of AM preservation techniques on EGF factor retention.

Preservation Treatment	Tissue Extract	Wash 1	Wash 2	Wash 3	Cumulative Wash	Retention Efficiency (%)
Fresh	0.977±0.030	0.079±0.011	0.057±0.019	0.029±0.003	0.165±0.032	83
Cryopreserved	0.540±0.033	0.267±0.007	0.140±0.006	0.064±0.011	0.471±0.024*	13
Dried	0.695±0.010	0.110±0.008	0.103±0.007	0.060±0.014	0.273±0.029†	61
Trehalose	0.689±0.082	0.044±0.021	0.025±0.010	0.017±0.001	0.086±0.032†	88
Raffinose	0.759±0.017	0.065±0.005	0.044±0.005	0.020±0.010	0.129±0.020†	83

AM samples were washed in saline solution for 10 minutes on three separate occasions. The washes were retained and concentrated prior to determining the EGF levels using a commercial ELISA kit (R & D Systems). Data are expressed as mean ± SEM based on three separate experiments.

* p<0.05 compared to fresh AM,

† p<0.05 compared to cryopreserved AM.

**Table 5:** The effects of AM preservation techniques on TGF-β1 factor retention.

Preservation Treatment	Tissue Extract	Wash 1	Wash 2	Wash 3	Cumulative Wash	Retention Efficiency (%)
Fresh	1.773±0.035	0.066±0.004	0.025±0.00 4	0.019±0.003	0.110±0.011	94
Cryopreserved	1.251±0.060	0.185±0.014	0.103±0.008	0.074±0.004	0.362±0.026*	71
Dried	1.587±0.116	0.131±0.009	0.062±0.008	0.023±0.00 3	0.216±0.020†	86
Trehalose	1.548±0.068	0.049±0.020	0.034±0.009	0.030±0.010	0.113±0.039†	93
Raffinose	1.368±0.046	0.056±0.007	0.038±0.002	0.011±0.004	0.105±0.013†	92

AM samples were washed in saline solution for 10 minutes on three separate occasions. The washes were retained and concentrated prior to determining the TGF-β1 levels using a commercial ELISA kit (R & D Systems). Data are expressed as mean ± SEM based on three separate experiments.

*p<0.05 compared to fresh AM,

† p<0.05 compared to cryopreserved AM.

**Figure 2:**
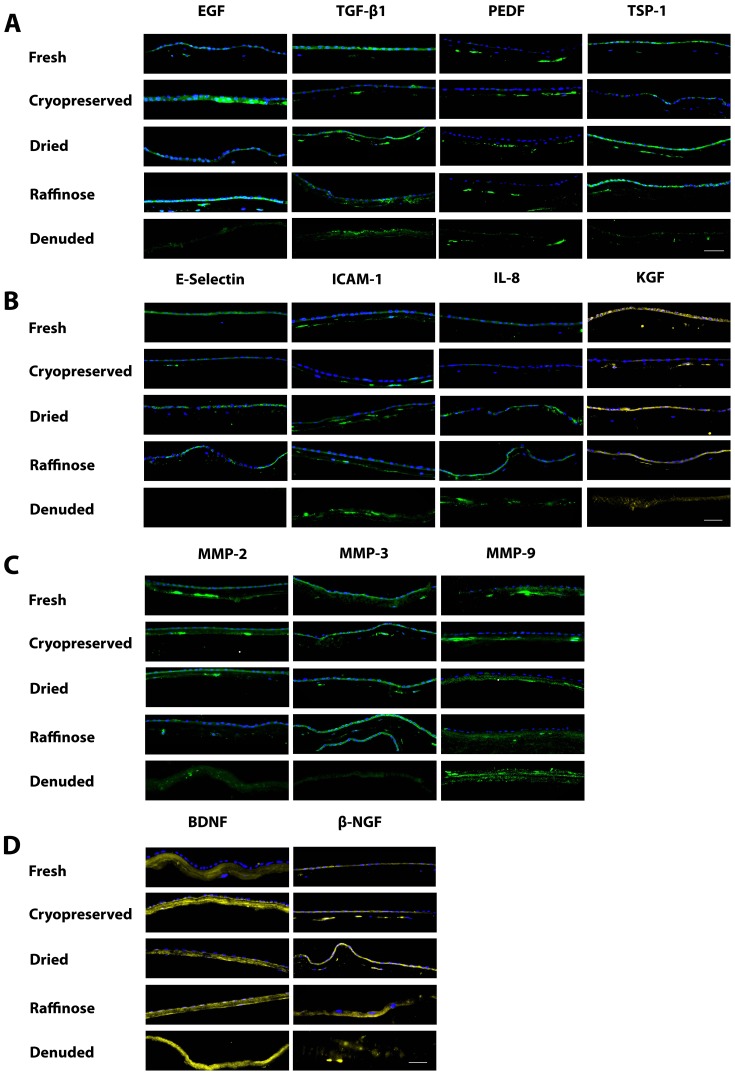
Expression of proteins with functions involved in ocular disease and wound healing in AM substrates. Proteins detected include growth factors and biomarkers (**A**) cell adhesion, cytokine and angiogenesis markers (**B**) metalloproteases and (**C**) neurotrophic factors (**D**). The collective pattern in staining demonstrates comparable levels of expression in trehalose and raffinose treated AM compared to fresh and increased expression compared to cryopreserved AM. Positive staining is represented as green or yellow and AEC nuclei were counterstained with DAPI (blue). Images shown are representative of triplicate experiments carried out on three donor membranes. Scale bar, 100 µm.

For *in situ* validation, 13 protein markers were selected from the 5 different factor cohorts described earlier and with functions potentially involved in ocular disease and epithelial wound healing (Figure 2). The collective pattern in staining demonstrated a decrease in protein detection in 77% (10/13) of markers in cryopreserved AM, compared to fresh AM (Table 6). The exceptions were the matrix metalloproteases (MMP)-2, MMP-9 and brain derived neurotrophic factor (BDNF), where staining intensities remained constant across the different preservation techniques as they were stromal in origin and mostly insoluble. Interestingly, staining for predominantly stromal-derived markers e.g. Pigment epithelium-derived factor (PEDF), MMP-2 and intercellular adhesion molecule (ICAM)-1, appeared more intense in the periphery of keratocytes (Figure 2). In dried AM, protein detection was comparable to fresh AM in 69% (9/13) of markers assessed, while comparable detection in raffinose treated AM was 85% (11/13) (Figure 2 and Table 6).

**Table 6:** A summary of protein expression levels of a panel of biochemical markers in AM substrates.

Marker	Function	Type	Fresh	Cryo	Dried	Raffinose
KGF	Angiogenesis	Epithelial	>75	>50	>75	>75
PEDF	Biomarker	Stromal	>75	>50	>50	>75
TSP-1	Biomarker	Epithelial	>75	>50	>75	>75
E-Selectin	Cell Adhesion	Epithelial	>75	>50	>75	>75
ICAM-1	Cell Adhesion	Stromal	>75	>25	>75	>75
IL-8	Cytokine	Epithelial	>75	>50	>50	>75
EGF	Growth Factor	Epithelial	>50	>50	>50	>75
TGF-β1	Growth Factor	Epithelial/Stromal	>75	>50	>75	>50
MMP-2	Metalloprotease	Epithelial/Stromal	>75	>75	>75	>50
MMP-3	Metalloprotease	Epithelial/Stromal	>75	>50	>75	>75
MMP-9	Metalloprotease	Stromal	>75	>75	>75	>75
BDNF	Neurotrophic Factor	Stromal	>50	>75	>50	>75
β-NGF	Neurotrophic Factor	Epithelial/cellular	>75	>50	>75	>75

Sections of AM were immunostained with their respective primary conjugates and counterstained with DAPI. Protein expression levels were determined from the resultant images and compared to expression fresh AM. Protein levels are represented as percentage total membrane staining.

*In situ* protein expression data supported SearchLight protein array data analysis demonstrating a significant decrease in protein expression in cryopreserved AM, particularly in epithelial-derived and soluble proteins. However drying and pre-treatment with raffinose prevented this protein loss, retaining beneficial factors more effectively, with little or no loss (Figure 2 and Table 6).

### Biochemical factor time release

**Figure 3:**
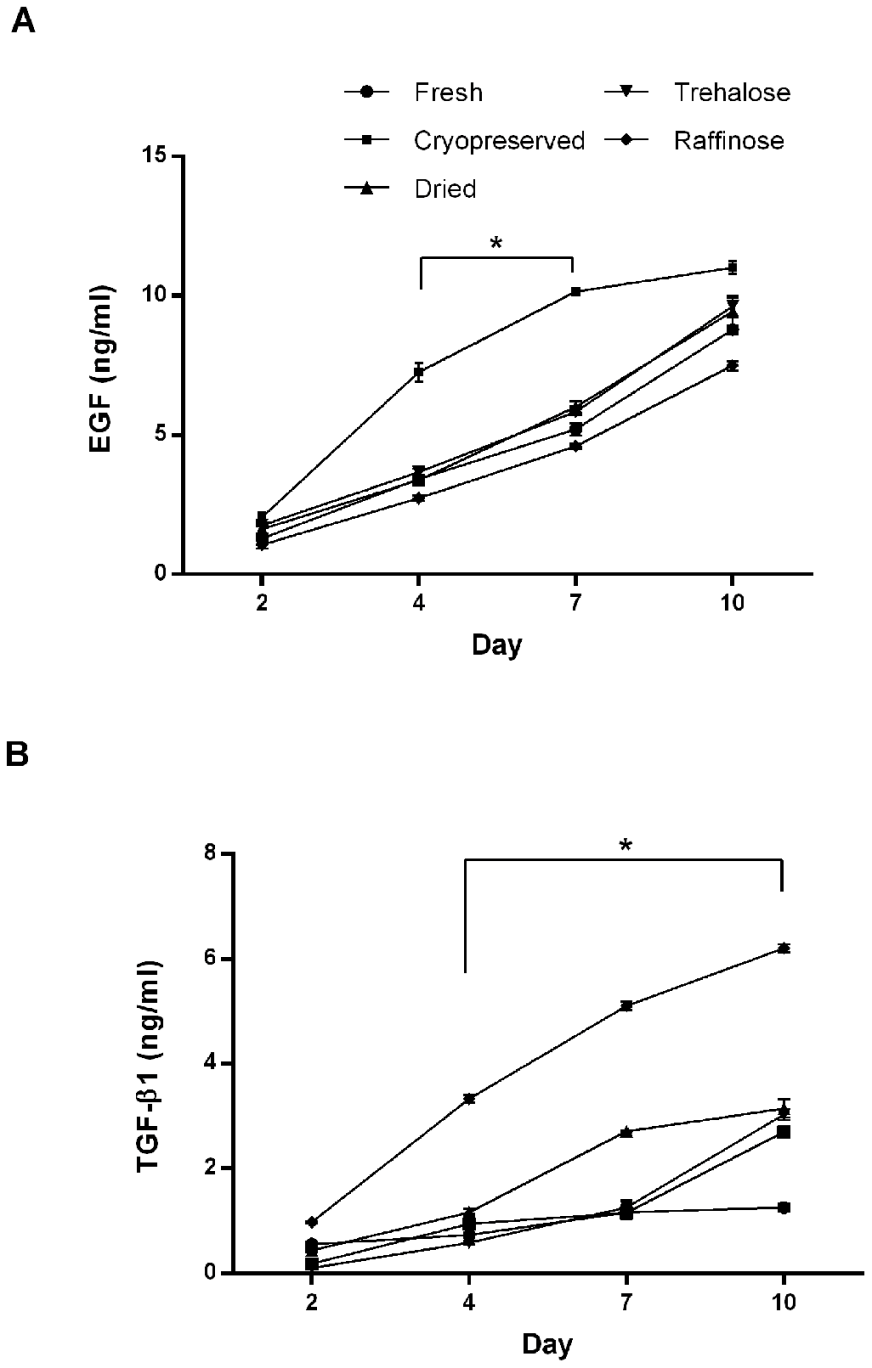
EGF and TGF-β1 biochemical factor release profiles from AM substrates preserved using different techniques. Preparations of AM were cultured over a 10 day period in PBS, using Scaffdex supports. Samples of PBS were taken at different time points and EGF (**A**) and TGF-β1 (**B**) levels were measured by ELISA. Release profiles demonstrate an immediate and time dependent release of both EGF and TGF-β1 from all of the AM substrates, over the 10 day culture period. Cryopreserved AM showed a significant increase (^*^p<0.05) in EGF release at days 4 and 7 compared to the sustained release from the remaining substrates at the equivalent time points. Conversely raffinose treated AM demonstrated a significant increase (^*^p<0.05) in TGF-β1 release at days 4, 7 and 10 compared to cryopreserved AM and the remaining substrates. Data are expressed as mean ± SEM based on six separate experiments.

Time release studies showed that there was an immediate and time dependent release of EGF and TGF-β1 in culture, over a 10 day period (Figure 3). The EGF profile showed a sudden increase in EGF release from cryopreserved AM at day 4 (7.26±0.33 ng/mL) and this then decreased over time. EGF release from trehalose and raffinose treated AM demonstrated a more sustained release over time with levels ranging from 1.75–9.62 ng/mL and 1.06–7.50 ng/mL respectively (Figure 3A)

TGF-β1 profiles showed a sustained time release from cryopreserved and trehalose treated AM with levels peaking at 2.70±0.10 ng/mL and 3.03±0.10 ng/mL at day 10. However TGF-β1 release from raffinose treated AM was sustained and amplified peaking at 6.20±0.08 ng/mL at day 10 (Figure 3B).

### Biochemical stability of amnion following preservation and storage

**Figure 4:**
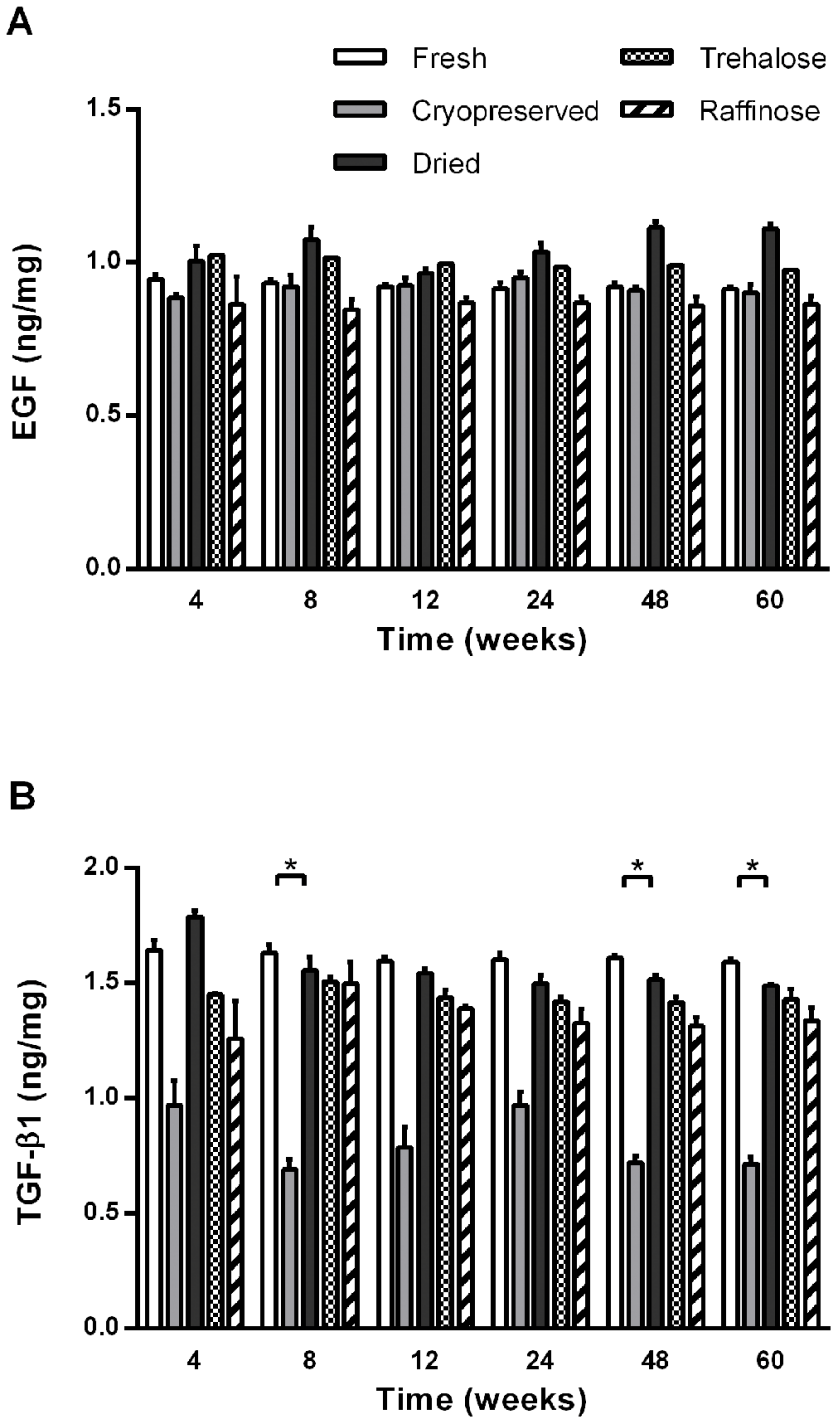
The effect of extended storage on EGF and TGF-β1 biochemical factor stability in AM substrates. EGF (**A**) and TGF-β1 (**B**) levels were measured by ELISA in AM substrates stored for extended periods of 4, 8, 12, 24, 48 and 60 weeks, away from direct light and at ambient temperature. No significant changes in EGF concentration were measured in any of the AM substrates following extended storage. The TGF-β1 profile illustrates biological variation between samples and a significant decrease (^*^p<0.05) in TGF-β1 levels in cryopreserved AM following 60 weeks of storage compared to 4 weeks. Data are expressed as mean ± SEM based on three separate experiments.

To investigate the stability of factors over long term storage, the levels of two significant wound healing factors were assessed sequentially over a period of 15 months. No significant changes in EGF concentration were observed in any of the substrates following preservation and storage. TGF-β1 concentrations appeared to vary between samples, illustrating biological variation. Results show a significant decrease in TGF-β1 in cryopreserved AM following extended storage periods (Figure 4).

### The indirect effect of preserved amniotic membrane substrates on cellular health

#### Proliferation

**Figure 5:**
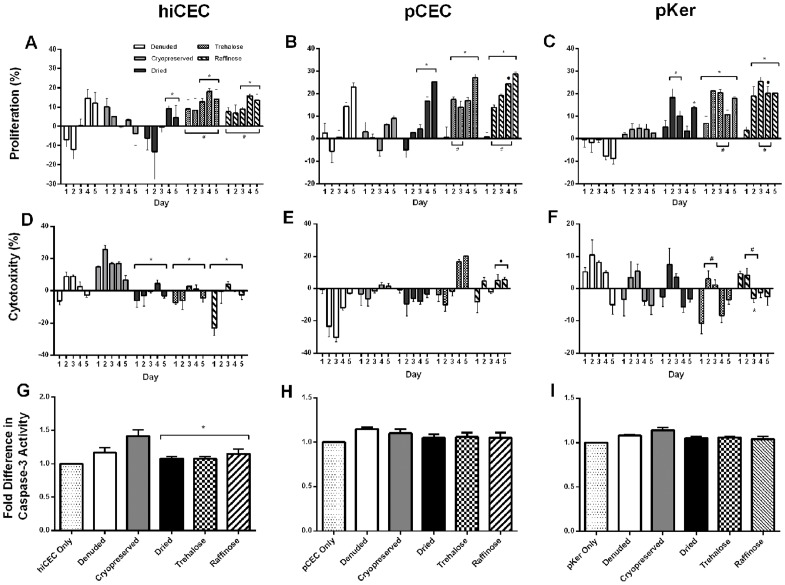
Proliferation, cytotoxic and apoptotic effects of AM substrates cultured indirectly with corneal epithelial cells. Levels were measured in cultures of hiCEC (A, D and G), pCEC (B, E and H) and pKer (C, F and I) by WST-1, LDH and caspase-3 assays. Cells were cultured with AM substrates over a 5 day period and changes in levels are relative to the previous day. Dried and AM substrates pre-treated with trehalose or raffinose stimulated the proliferation of pCEC and pKer, and exerted negligible cytotoxic or apoptotic effects compared to denuded or cryopreserved AM. Data are expressed as mean ± SEM based on three separate experiments. ^*^p<0.05 increase or decrease compared to cells cultured with cryopreserved AM; ^#^ p<0.05 increase or decrease compared to cells cultured with dried AM; ^•^ p<0.05 increase or decrease compared to cells cultured with raffinose treated AM.

Indirect culture of cryopreserved AM with hiCEC induced a stable decrease in proliferation over the 5 day culture period, compared to cells cultured without AM (Figure 5A). Similarly poor proliferation rates were observed when cultured with pCEC were used (Figure 5B). Although denuded and dried AM induced a dramatic initial drop in proliferation in the initial 48 hour period in both hiCEC and pCEC, a dramatic increase in proliferation was observed with proliferation rates of 15% and 9% in hiCEC and 14 and 17% in pCEC at day 4. In contrast, trehalose and raffinose treated AM consistently promoted proliferation over the entire culture period, and at comparable rates, with levels peaking at 18 and 16% in hiCEC at day 4 and 27 and 29% in pCEC at day 5 (Figure 5A and B).

The effect of AM substrates on primary keratocyte (pKer) proliferation was tested in a similar manor. Indirect culture showed a progressive increase in proliferation in the presence of dried, trehalose and raffinose treated AM compared to cryopreserved AM, with trehalose and raffinose AM promoting greater rates of growth. Increasingly negative proliferation rates were observed with denuded AM (Figure 5C).

#### Cytotoxicity

Indirect culture of cells with dried, trehalose and raffinose treated AM demonstrated generally reduced cell death in both hiCEC and pCEC (Figure 5D and E) validating mirror proliferation profiles (Figure 5A and B). Cryopreserved AM, and to a lesser degree denuded AM, exerted a greater cytotoxic effect in hiCEC cultures with levels reaching 25% and 9% at day 2, respectively (Figure 5D). This data supports the poor proliferation rates observed in Figures 5A and B. Interestingly, cytoxicity was greatly reduced in pCEC cultured with denuded AM suggesting pCEC are less sensitive to cytotoxic insults, which was probably epithelial in origin and removed with denuding (Figure 5E). All substrates, particularly denuded AM promoted cytotoxicity in pKer during the initial culture period but levels continued to decrease over time (Figure 5F).

#### Apoptosis

Indirect culture of dried preparations with CEC or pKer promoted no significant increases in caspase-3 activity compared to cell only controls (Figure 5H and I). Marginal increases in caspase-3 activity were observed with cryopreserved and denuded AM across all cell types except cryopreserved AM significantly (p<0.05) increased caspase-3 activity when indirectly cultured with hiCEC (0.45 fold) (Figure 5G).

### The direct effect of preserved amniotic membrane substrates on cellular health

#### Proliferation

**Figure 6:**
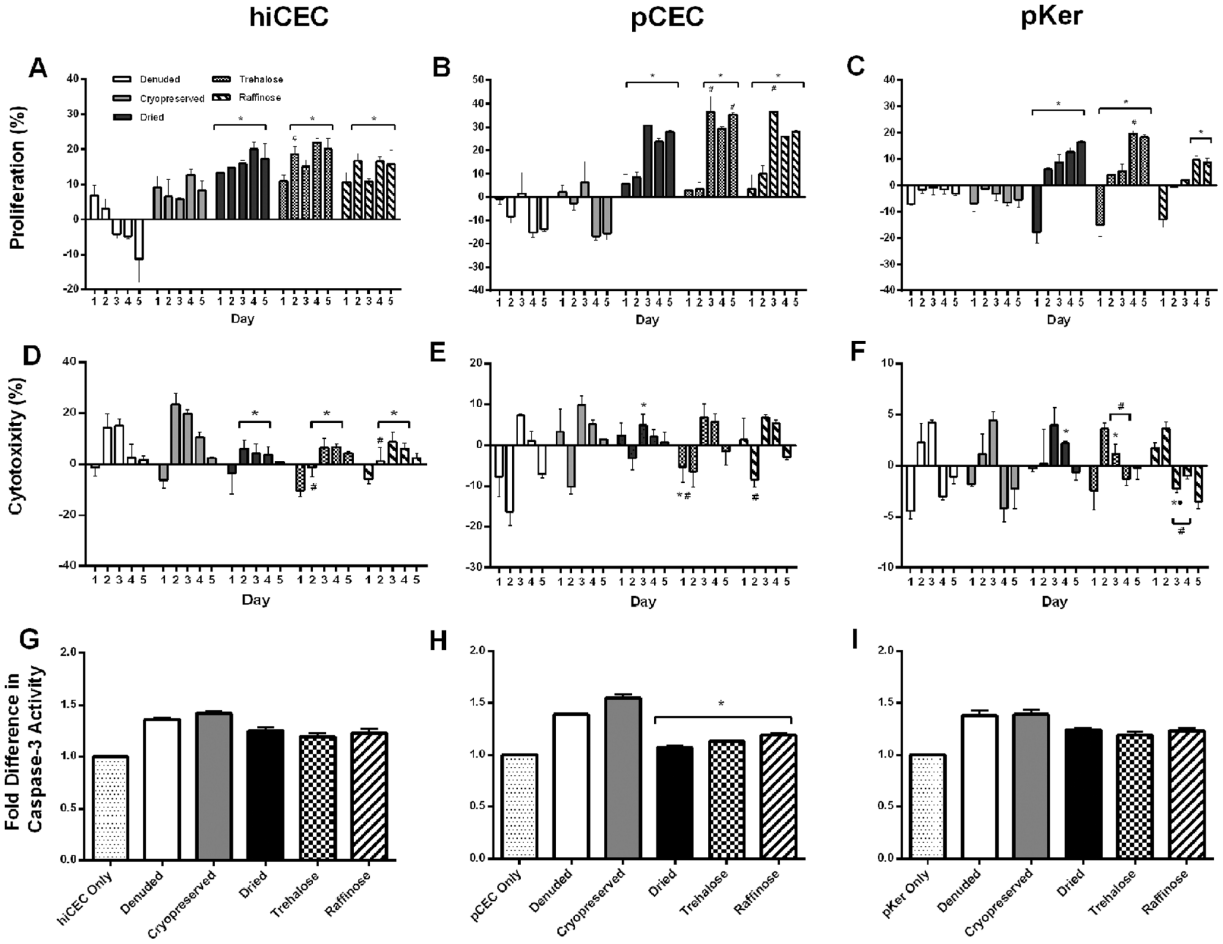
Proliferation, cytotoxic and apoptotic effects of AM substrates cultured directly with corneal epithelial cells. Levels were measured in cultures of hiCEC (**A**, **D** and **G**), pCEC (**B**, **E** and **H**) and pKer (**C**, **F** and **I**) by WST-1, LDH and caspase-3 assays. Cells were cultured with AM substrates over a 5 day period and changes in levels are relative to the previous day. Dried and AM substrates pre-treated with trehalose or raffinose stimulated proliferation of CEC with levels comparable or greater to indirect cultures and reduced the proliferation of pKer. Denuded and cryopreserved substrates produced a negative effect on proliferation across all the cell types. The overall cytotoxic apoptotic effects were greater than in cells cultured indirectly and more pronounced when cultured with denuded and cryopreserved substrates. Data are expressed as mean ± SEM based on three separate experiments. ^*^p<0.05 increase or decrease compared to cells cultured with cryopreserved AM; ^#^ p<0.05 increase or decrease compared to cells cultured with dried AM; ^•^ p<0.05 increase or decrease compared to cells cultured with raffinose treated AM.

Direct culture promoted a greater and comparable increase in proliferation in both hiCEC and pCEC using dried, trehalose and raffinose treated AM (Figure 6A and B), compared to indirect culture (Figure 5A and B). This effect was most pronounced in pCEC with the maximum proliferation rates of 28–35% at day 3 observed with trehalose and raffinose treated AM (Figure 6B). Although direct culture improved proliferation rates of hiCEC with cryopreserved AM, proliferation rates were not improved in pCEC. While indirect culture with denuded AM marginally increased hiCEC proliferation and to a greater extent pCEC proliferation post day 3, direct culture of both cell types with denuded AM induced a considerable negative proliferative effect (Figure 6A and B).

Conversely, direct culture of pKer with all membrane preparations resulted in considerable negative effects on proliferation, supporting the evidence that AM inhibits pKer growth[49], [50]. The negative growth effect was sustained with denuded and cryopreserved AM, but was overpowered with dried membranes (Figure 6C), most likely due to a greater retention of growth factors. However proliferation was reduced (18% for any time point) compared to indirect pKer cultures (Figure 5C).

#### Cytotoxicity

Direct culture of all samples types showed a moderate increase in cytoxicity after the first (hiCEC) and second (pCEC) day, with the greatest cytoxicity observed with cryopreserved AM and least so in dried, trehalose and raffinose treated AM (Figure 6D and E). The increase in cytoxicity was greater and more evident with hiCEC compared to indirect. The cytotoxic effect of direct culture with dried AM peaked at no more than 5%, and decreased post day 3 (Figure 6D and E).

Cytoxicity in direct cultures of both CEC cell types appeared to follow a pattern, which is most clearly seen in hiCEC, in that following an initial reduced cytoxicity on the first day (first two days and at a greater reduction for pCEC) a sharp increase in cytoxicity appeared to consistently occur on day 3 which then decreased over the remaining culture period (Figure 6D and E). This pattern was also similar but more pronounced when pKer were cultured with the various AM preparations. For direct culture of pKer, levels did not exceed 5% and decreased to nominal levels post 3 days in culture (Figure 6C). Culture with denuded AM showed the most pronounced cytotoxic effect at day 3 (Figure 6C). Direct cultures with AM preparations showed similar cytotoxic profiles to indirect culture with cytotoxicity decreasing over the 5 day culture period and levels did not exceed 5% (Figure 6C).

#### Apoptosis

Cryopreserved AM significantly (p<0.05) increased caspase-3 activity when directly cultured with hiCEC (0.4 fold), CEC (0.6 fold) and pKer (0.4 fold), compared to cells alone, following five days in culture (Figure 6G–I). This suggests cryopreserved AM, which is comparable to conventional intact AM, induces cell death through apoptosis. Similarly denuded AM significantly (p<0.05) increased caspase-3 activity compared to control, when directly cultured with hiCEC, pCEC and pKer (0.4 fold each). Although some increases in caspase-3 activity were observed for the remaining cell/dried substrate cultures, these were non-significant (Figures 6G–I).

### Wound healing

**Figure 7:**
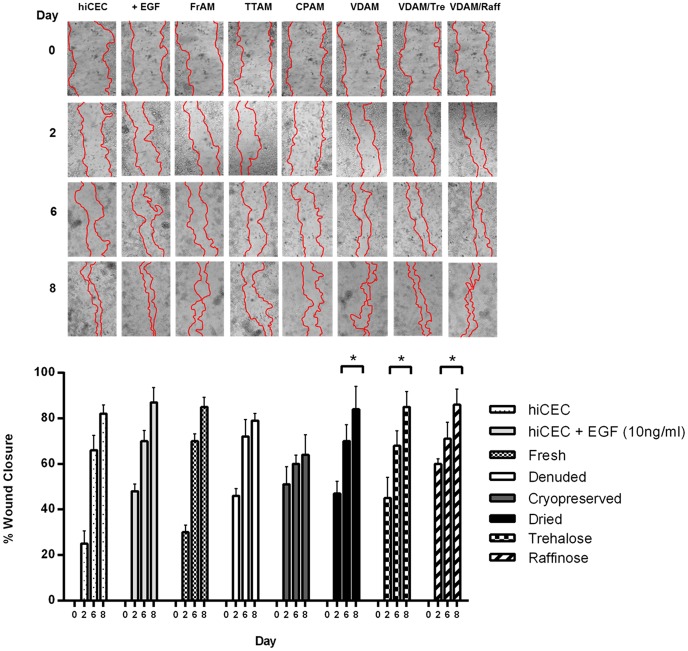
The indirect effect of AM substrates on hiCEC re-epithelialisation following injury. Re-epithelialisation of hiCEC in response to the substrates was assessed using a scratch assay and the wound areas were measured from micrographs taken at day 0, 2, 4, 6 and 10 days (**A**). Wound areas at days 2, 6 and 8 were compared to wound areas at day 0 using ImageJ software and percentage wound closure rates were calculated (**B**). Cell migration and re-epithelialisation was evident in all of the wounds over an 8 day period. Denuded and cryopreserved AM substrates demonstrated no additive effect on re-epithelialisation compared to hiCEC cultured without AM. Dried, trehalose and raffinose treated AM significantly promoted re-epithelialisation and wound healing compared to hiCEC cultured with cryopreserved AM. Data are expressed as mean ± SEM based on three separate experiments. ^*^p<0.05 increase in re-epithelialisation compared to corresponding time points in cells grown with cryopreserved AM.

To evaluate the effects of the AM substrates on re-epithelialisation following injury, scratch wound healing assays were performed and wound closure rates calculated at days 2, 6 and 8 compared to control (day 0) and in the presence of hiCEC indirectly cultured with the different AM preparations (Figure 7A). Results showed that although all samples demonstrated wound healing up to 8 days and beyond, fresh, denuded, and cryopreserved AM showed no improvement over hiCEC alone (no amnion control). Dried, trehalose and raffinose treated AM significantly improved wound healing with wound closure rates of 84–86% at day 8 compared to 64% in hiCEC cultured with cryopreserved AM (Figure 7B).

## Discussion

In recent years freeze-dried AM has become an alternative to cryopreserved AM as a substrate for stem cell expansion[14], [29] and as a conjunctival replacement in pterygium surgery[14]. In addition dried AM preparations, cross-linked with glutaraldehyde, have been used as a primary treatment for corneal perforations, indicating its increased usefulness over cryopreserved AM in certain clinical situations[51]. This may reflect easier surgical handling of the dried tissue, which can be glued rather than sutured onto the ocular surface[30]. Evidence suggests that dried AM may be advantageous in a number of clinical conditions, for instance as an inlay graft for persistent epithelial defects or as a patch for the entire ocular surface in acute burns. Animal model studies have indicated that freeze-dried AM is at least as effective as cryopreserved AM as a substrate for ocular surface reconstruction[52]. Besides offering clinical advantages, dried AM eliminates the need for temperature-controlled transportation, significantly reducing expenditure.

To minimise the effects of freeze damage, we pre-treated AM with lyoprotectants prior to drying. Our SEM images show that while cryopreservation and drying without lyoprotectants are highly destructive to cellular integrity, our trehalose or raffinose treated AM preparations exhibited less visible structural damage and appear structurally akin to fresh AM. This was supported by TEM images showing that dried AM without pre-treatment had a more condensed collagen network, and the stromal and epithelial layers appeared thinner following the drying process. While the visual quality of reconstituted lyophilised AM has been reported to be poor in comparison to fresh AM and cryopreserved AM[53], our studies found no obvious differences in quality. We attribute these findings to pre-treatment with a saccharide lyoprotectant and the elimination of a pre-freeze step allowing preservation of cellular and matriceal integrity.

In addition to the structural differences observed, our study confirmed that all preservation methods resulted in the loss of biological factors from AM.

In 2009, Rodriguez-Ares and colleagues published similar results to our own findings. They analysed the biochemical profiles of AM preparations, concluding that the levels of biochemical factors associated with wound healing were decreased in lyophilised AM compared to cryopreserved, but these were not significant[32]. The biochemical profile of our raffinose treated AM showed an increase in 71% (32/45) of the total factors analysed compared to cryopreserved AM, and an increase in 64% (29/45) of factors in dried AM compared to cryopreserved AM (Table 3). This contradicts previous research findings suggesting diminished growth factor levels in lyophilised compared to cryopreserved AM[54]. This demonstrates that trehalose and raffinose stabilise the tissue, enabling a more physiological biomaterial to be produced. In the future this may contribute to improved clinical outcomes.

Differences in preservation methods do not solely explain the variation in AM factor levels, which may also be influenced by gestational age, donor age, differences in handling techniques and the location of the sample's origin within the donor tissue[26], [54]. However, our immunofluorescence data confirm our finding that lyoprotection of AM with trehalose or raffinose prevents loss of soluble biological factors and this is consistent with the protein array data. In contrast, an immunohistochemical analysis of AM extracellular matrix molecules has indicated reduced intensity following drying compared to cryopreservation[55]. This may be attributed to radiation damage.

Assuming that the retention of key growth factors in AM preparations is germane to its effectiveness in some clinical situations, the rate of release of these factors may also be significant. Ideally, AM would secrete its factors over a prolonged period and not its whole reserve instantaneously. Our assays show that as well as improved factor retention, the release of EGF from trehalose or raffinose treated AM was more sustained than cryopreserved AM over a 10 day period suggesting that the immediate release from cryopreserved was attributable to the freeze-thaw damage. This indicates that optimised trehalose and raffinose treated AM will have both increased factor content and prolonged release potentially improving the overall clinical effectiveness of AM over cryopreserved AM.

Cellular health assays supported this showing trehalose and raffinose treated AM substrates promote significant increases in corneal epithelial and keratocyte proliferation, with reduced overall cytotoxicity and apoptotic levels, compared to denuded and cryopreserved AM in both direct and indirect cultures. Similarities between cryopreserved and denuded AM further suggest that this phenomenon is explained by the removal of essential cell-based growth factors essential for promoting wound healing, rather than as a result of drying *per se*.

Increased wound closure rates were observed in hiCEC co-cultures with the optimised trehalose and raffinose treated AM substrates compared to cryopreserved and fresh. This may be attributed to the bioavailability of wound healing factors e.g. EGF, HGF, PDGF and TGF-β retained in the tissue by our optimised preservation technique. Growth factors are essential for the regulation of cellular processes including growth, proliferation and differentiation. If certain factor levels are diminished or absent this would have a detrimental effect on these processes and subsequent wound healing.

## Conclusions

In this study, we have shown that the saccharide lyoprotectants trehalose and raffinose improve the quality of dried AM by maintaining its structural and biochemical properties over extended periods. This improved stability will allow our optimised dried AM substrate to be stored and transported without the need for freezing, reducing costs and allowing it to be used as an ocular surface dressing outside modern hospital settings. In the absence of availability of optimised drying procedures our work indirectly supports the use of denuded AM, using an optimised procedure[45] is more effective than conventional intact cryopreserved AM.
